# Circumpolar permafrost maps and geohazard indices for near-future infrastructure risk assessments

**DOI:** 10.1038/sdata.2019.37

**Published:** 2019-03-12

**Authors:** Olli Karjalainen, Juha Aalto, Miska Luoto, Sebastian Westermann, Vladimir E. Romanovsky, Frederick E. Nelson, Bernd Etzelmüller, Jan Hjort

**Affiliations:** 1 Geography Research Unit, University of Oulu, Oulu, Finland; 2 Department of Geosciences and Geography, University of Helsinki, Helsinki, Finland; 3 Finnish Meteorological Institute, Helsinki, Finland; 4 Department of Geosciences, University of Oslo, Oslo, Norway; 5 Geophysical Institute, University of Alaska Fairbanks, Fairbanks, Alaska, USA; 6 Department of Cryosophy, Tyumen State University, Tyumen, Russia; 7 Department of Geography, Environment, and Spatial Sciences, Michigan State University, East Lansing, Michigan, USA; 8 Department of Earth, Environmental, and Geographical Sciences, Northern Michigan University, Marquette, Michigan, USA

**Keywords:** Cryospheric science, Climate and Earth system modelling, Natural hazards

## Abstract

Ongoing climate change is causing fundamental changes in the Arctic, some of which can be hazardous to nature and human activity. In the context of Earth surface systems, warming climate may lead to rising ground temperatures and thaw of permafrost. This Data Descriptor presents circumpolar permafrost maps and geohazard indices depicting zones of varying potential for development of hazards related to near-surface permafrost degradation, such as ground subsidence. Statistical models were used to predict ground temperature and the thickness of the seasonally thawed (active) layer using geospatial data on environmental conditions at 30 arc-second resolution. These predictions, together with data on factors (ground ice content, soil grain size and slope gradient) affecting permafrost stability, were used to formulate geohazard indices. Using climate-forcing scenarios (Representative Concentration Pathways 2.6, 4.5 and 8.5), permafrost extent and hazard potential were projected for the 2041–2060 and 2061–2080 time periods. The resulting data (seven permafrost and 24 geohazard maps) are relevant to near-future infrastructure risk assessments and for targeting localized geohazard analyses.

## Background & Summary

The functioning of Arctic environments is fundamentally dependent on ground temperature conditions^1^. Terrestrial permafrost, defined as soil or bedrock at or below 0 °C for at least two consecutive years^2^, is a natural phenomenon closely related to the thermal dynamics of northern landscapes. For human activity, soils in a frozen state are important because they support, and in certain regions allow for development of infrastructure. Recently observed rapid changes demonstrate that the Arctic region is showing particular sensitivity to climate change^1,3^. Projected climate warming is expected to increase permafrost temperatures and promote thaw, exerting a threat to human activities and infrastructure in permafrost regions^4–6^. Melting of ground ice in near-surface permafrost can lead to ground subsidence in areas with ice-rich sediments^7–9^.

A consensus has formed that the geographic extent of permafrost will decrease substantially by the end of 21^st^ century (e.g., refs^10–13^), although the magnitude of change is mediated by local factors. Snow, vegetation, soil texture and moisture, and topography-controlled factors (e.g., exposure to solar radiation and hydrology) regulate the flow of heat between the atmosphere and ground, resulting in a complex set of processes governing the ground thermal regime^14–17^. Consideration of local environmental factors in ground temperature modelling is therefore crucial, especially in topographically heterogeneous areas associated with pronounced fine-scale variability in, for example, microclimate and soil thermal and hydrogeological properties^18–20^.

**Figure 1:**
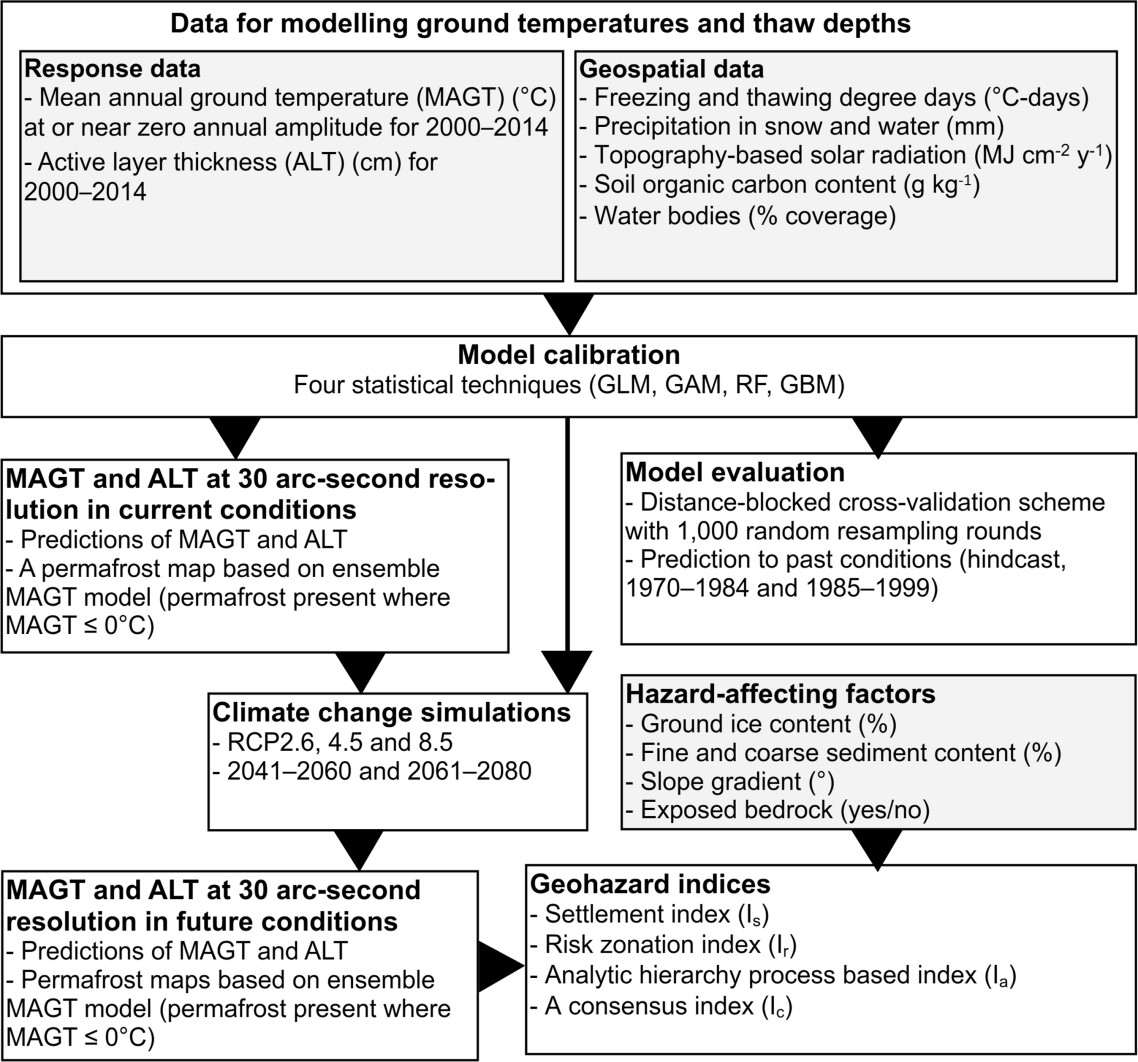
Schematic depiction of modelling data, model calibration and evaluation, and resulting data layers employed in this study. Observational data on mean annual ground temperature (MAGT) and active layer thickness (ALT), together with geospatial data, were used to calibrate statistical models using generalized linear models (GLM), generalized additive models (GAM), random forest (RF) and generalized boosted models (GBM). The modelling outputs were predictions of MAGT and ALT in current conditions and a derived map for suitable conditions for permafrost (i.e., predicted MAGT ≤ 0 °C). Models were evaluated with a distance-blocked cross-validation scheme that accounted for spatial autocorrelation, and with past MAGT and ALT observations. Three emission trajectories (RCPs = representative concentration pathways) were implemented to simulate MAGT and ALT under future conditions. Finally, additional geospatial predictors affecting local hazard potential were used with MAGT and ALT predictions to formulate geohazard indices.

In this study, the potential of permafrost occurrence in changing climate was estimated with two inherently connected subsurface properties: mean annual ground temperature (MAGT) at or near the depth of zero annual amplitude (DZAA) and the thickness of the seasonally thawed layer (active layer thickness, ALT) above permafrost. We first calibrated statistical models to predict present day (2000–2014) MAGT and ALT in land areas of the Northern Hemisphere north of 30 degrees latitude (Fig. 1). Permafrost maps were then derived from MAGT predictions (Data Citation 1). The predictive performances of the models were evaluated with distance-blocked cross-validation and a hindcasting approach using past measured ground temperatures from the 1970–1984 and 1985–1999 periods. Next, we predicted climate-warming-induced changes in MAGT and ALT in the future (2041–2060 and 2061–2080) under three greenhouse gas emission trajectories (Representative Concentration Pathways, RCPs 2.6, 4.5 and 8.5; refs^21,22^). Finally, taking into account additional factors that affect local hazard potential (e.g., ground ice content and soil properties), we formulated four high-resolution hazard indices (settlement index, risk zonation index, analytical hierarchy process based index, and the consensus of these three) delineating areas of varying potential for damage to infrastructure related to near-surface permafrost degradation (Data Citation 1). The authors used these data products to quantify infrastructure hazards and risks subject to near-surface permafrost degradation^23^. This Data Descriptor provides a detailed description of methods and data products presented therein.

The demand for improved understanding of permafrost dynamics in a changing climate has been recognized on the international level^4,6,24,25^. Moreover, many recent national-scale studies suggest that risks to infrastructure related to permafrost degradation will increase over the next decades^5,7,26–28^. There is, however, a deficit of high-resolution large-domain assessments. Encompassing both a near-global extent and local-level resolution of analysis these data products provide insights into near-surface permafrost dynamics and the spatio-temporal development of thaw-related hazards.

## Methods

### MAGT data

**Figure 2:**
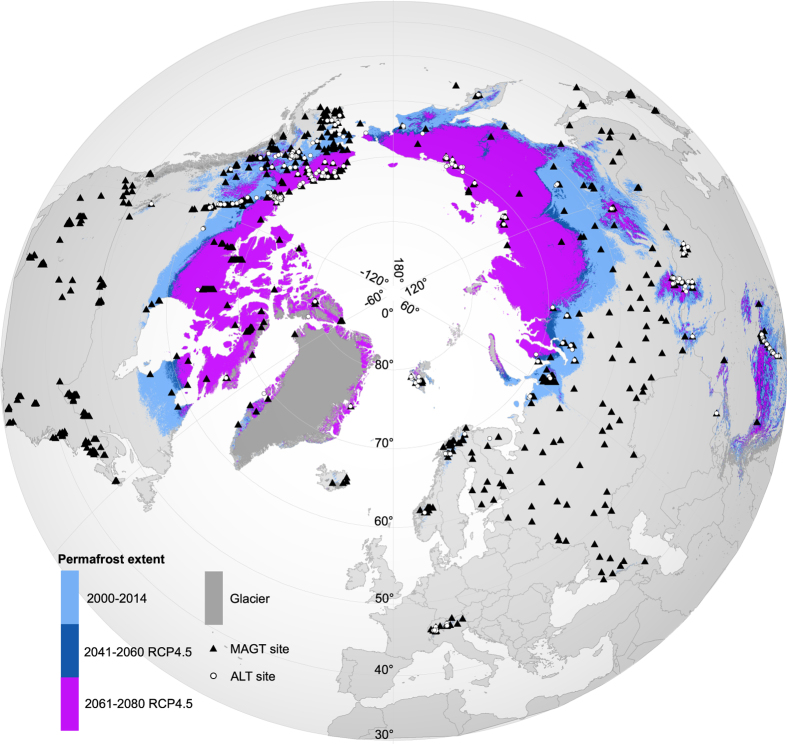
Mean annual ground temperature (MAGT, n = 797, black symbols) and ALT observation sites (active layer thickness, n = 303, white symbols) in the Northern Hemisphere north of 30°N. Modelled extent of near-surface permafrost occurrence, (predicted MAGT ≤ 0 °C), undifferentiated by continuity zone is shown for the present (2000–2014) and future (2041–2060 and 2061–2080) periods under a moderate climate-forcing scenario RCP4.5 (representative concentration pathways). In certain areas, especially in northwestern Russia, thaw of near-surface permafrost is projected to progress rapidly between the two future periods (dark blue zone), whereas in many other locations the change is minimal owing to, for example, the altitudinal cooling effect of a topographical barrier. World Borders dataset is distributed under CC BY-SA 3.0 license (https://creativecommons.org/licenses/by-sa/3.0/) on http://thematicmapping.org/downloads/world_borders.php.

The MAGT dataset is built upon the Global Terrestrial Network for Permafrost (GTN-P) database^29^ (gtnpdatabase.org). A substantial part of the GTN-P inventory consists of data from the Thermal State of Permafrost (TSP) Snapshot Borehole Inventory^30,31^, compiled during the fourth International Polar Year^29^. The GTN-P database has been updated regularly, with both inclusion of new boreholes and upkeep of ongoing observation data for existing study sites. We made use of these updated data to add observations into our dataset and to calculate MAGT values for boreholes without a value in the original TSP dataset. MAGT observations around 0 °C (especially between −2 and 0 °C), however, were overrepresented in these datasets. Therefore, we expanded the GTN-P data by including MAGT observations within and beyond the permafrost domain from additional sources^32–35^ (Data Citations 2, 3, 4, 5, 6, 7, 8, 9, 10) (Supplementary Table 1). Warm (MAGT > 0 °C) observations were a crucial addition in order to address the ground thermal regime in future climate conditions, i.e., to ascertain that calibrated model functions hold in cases when permafrost thaws and MAGT rises above 0 °C. Thus, there is no need to extrapolate the models outside the range of model calibration data. In this way, we were able to compile a spatially and environmentally more comprehensive database of 797 observations for land areas north of 30°N (Fig. 2).

Ideally, the MAGT observations should: (1) experience minimal short-term temperature fluctuations; (2) be representative of the same time period; (3) be free of disturbance, such as recent fires, large water bodies, or anthropogenic or geothermal heat sources in their immediate vicinity; and (4) have an adequate spatial precision to match with GIS and remote sensing based geospatial data. Not all the observations met the conditions stated above and some exclusions were necessary, based on the following criteria:

#### (In)variability of ground temperature

Spatial and temporal comparability of ground temperatures both inside and outside the permafrost domain was ascertained by using values at or near DZAA where diurnal, synoptic and seasonal variations are non-existent or minimal, i.e., less than 0.1 °C (ref.^36^). Although ground temperatures at DZAA are virtually stable annually, long-term and inter-annual variations may occur^37^. Thus, we delimited our datasets to a recent 15-year period (2000–2014); both to limit the effect of long-term trends and to assure the availability of enough multi-year observations needed to rule out inter-annual variability. Owing to possible regional climatic trends, the observations representing only the first or last year(s) of the study period may have differed from full period averages but we considered this hypothetical bias to have been non-systematic and inconsequential to the results.

When DZAA was not reported in the source data, we used the available ground temperatures to determine a MAGT value with intra-annual temperature variation less than 0.1 °C. If numerical data were not available, we used temperature-depth curves (‘trumpet curves’) drawn from year-round measurements to record a MAGT at a depth with no perceivable annual fluctuation^31^. When DZAA was reportedly not reached, we calculated MAGT from time-series data assuming that averaged year-round temperature measurements could be used to derive more representative annual means than single measurements. For example, Ref.^38^ concluded that the direction and magnitude of regional trends measured at 3.2 m depth in the Roshydromet data (see Supplementary Table 1) were similar to those measured in deeper boreholes across the Arctic. Therefore, we considered such measurements suitable for assessing average ground thermal conditions over the studied period, although in some conditions possible short-term reversals in air temperature trends might have affected shallow and deep MAGTs at different times. However, we excluded all observations less than 2 meters below the surface if not specifically verified to represent the temperature at DZAA (one observation site).

Some of the data without information on DZAA were based on a single temperature measurement. In these cases, we chose a value at or closest to 15 m below the ground surface. This estimate was based on the assumption that at this depth seasonal temperature variation under most conditions is non-existent or marginal. Notwithstanding the substantial spatial variation of DZAA globally and regionally, it usually occurs between 10–15 m^36,39^, 10–20 m^37,40^, 15–20 m^8^, and within 20 m^41^ of the surface.

#### Disturbance

For all raw data, we performed a careful examination of temperature-depth curves and recorded values to eliminate any disturbed observations. We also considered the probes used for certain non-permafrost area measurements without information on DZAA. If a probe designed to measure water temperature was used where the 15-meter depth was above the surface of ground water level, observations were considered invalid. Whenever it was evident from source data or after careful examination of borehole location that any disturbance had an obvious effect on the ground thermal regime, we excluded the observation. These rare omissions occurred when a borehole was positioned in an anomalous location, e.g., in an area of recent forest fire or on a small island affected by the heat in lake bodies^41,42^.

#### Location precision

The grid-cell size of the geospatial data (30 arc seconds, ca. 932 × 932 m at the Equator, ca. 932 × 466 m at 60°N latitude) was the determining factor in assessing adequate location precision for the MAGT observations. A minimum of three decimal places precision (WGS 1984, ca. 111 m at the Equator) was required. As a consequence of the poleward narrowing of grid cells, this is ca. 56 meters of longitudinal precision at 60°N latitude. For observations with lower precision, more precise geopositioning information was obtained from previous studies and original data sources. We made an exception with two vital additional datasets covering large areas of Japan^43^ and Russia^44^, wherein a precision of two decimal places (ca. 560 meters of longitudinal precision at 60°N latitude) was accepted.

### ALT data

The Circumpolar Active Layer Monitoring Network (CALM) data included in the GTN-P database^29^ comprise the basis for ALT data used in this study. The ALT measurements are derived either mechanically by probing in grids or transects, or as single-point depth values from thaw tubes or soil temperature profiles^45^. Grids and transects include multiple individual measurements from which a mean value is calculated to represent ALT at a site^46^. In addition, we utilized other available data sources^47^ (Data Citations 6, 10, 11, 12, 13, 14, 15, 16, 17, 18, 19) (Supplementary Table 2) to compile a dataset of 303 ALT measurement sites across areas underlain by permafrost.

The comparability of ALT observations was ascertained by using measurements representative of the maximum annual thaw i.e., probing conducted at the end of a thawing season^16,45^. Because a large proportion of ALT measurements are conducted on grids that encompass areas from one hectare to one square kilometer, a lower minimum location precision of an arc minute i.e., ca. 926 m longitudinal precision at 60°N latitude was adopted. While acknowledging the potential high spatial variability of ALT over short distances^48,49^ we did this to avoid omitting a large part of the observations.

When an ALT site contained observations from several years, available values were averaged. A few single measurements were also included, although their representativeness for the whole period is likely to be relatively weak. They were, however, crucial in order to cover a maximum range of environmental conditions. We excluded any documented anomalous measurements from the calculations to ascertain unbiased observations. Omissions consisted of post-fire measurements, early summer measurements (probing performed before maximum thaw) and imprecisely reported depths including > or < signs.

If two or more MAGT or ALT observations occupied the same 30 arc-second grid cell, we limited the observations to the one in line with the criteria described above. Whenever available, a multiyear record of MAGT or ALT was preferred over a single observation or one-year-long measurement. In cases where no observation exhibited superior quality over the other, we chose the median value.

### Geospatial environmental data

We selected physically relevant geospatial data based on literature^14,16,17,40^, characterizing climate, soil, water bodies and topographic properties to be used as predictors in the statistical models to explain circumpolar MAGT and ALT variation (Table 1). The environmental data layers were clipped and sampled to the same spatial resolution (30 arc seconds) and extent (north of 30°N latitude) in geographic WGS 1984 projection. All data processing was conducted in ArcGIS 10.3 (ref.^50^).

We used the WorldClim data^51^ to derive predictors of climate conditions, i.e., air temperature and precipitation. The climate surfaces have been produced by interpolating climate parameters recorded at meteorological stations to 30 arc-second resolution using elevation, latitude and longitude as independent variables^51^. The temporal representativeness of the data (1950–2000) did not match the ground thermal data, and we first used the Global Meteorological Forcing Dataset for land surface modelling^52^ at a 0.5-degree resolution to account for changes in the climatic parameters between the two periods. After the GMFD data had been processed to represent monthly temperatures and precipitation sums for 2000–2014 (as well as for 1970–1984 and 1985–1999 for model evaluation, see Technical Validation), it was resampled to the 30 arc-second resolution using a nearest-neighbor method. Locally smoothed (3 × 3 pixel moving average) differences between the GMFD and WorldClim data were used to adjust the data into the desired periods.

Climate conditions for 2041–2060 and 2061–2080 were considered by using the 18-model ensemble projections from the Coupled Model Inter Comparison Project phase 5 archive^53^ included in the WorldClim data^51^. The downscaled and bias-corrected climate projections represented future conditions under our chosen three RCPs (RCP2.6, RCP4.5 and RCP8.5), wherein numerated values are global end-of-century radiative forcing estimates^21^ (W/m^2^). Similarly to the adjusted baseline climate data, these projections have absolute temperature and precipitation values based on which the following climatic predictors were calculated.

Monthly average air temperatures and precipitation sums were used to calculate freezing and thawing degree-days (FDD and TDD, °C-days) and annual precipitation (mm) in snow (precipitation sum for months with mean air temperature below 0 °C) and rain (months above 0 °C). FDD and TDD were produced as sums of monthly air temperatures below and above 0 °C, respectively, multiplied with averaged 30-day months. It has been shown that using monthly rather than daily meteorological data to derive FDD and TDD introduces errors of less than 5% for most high-latitude land areas^54^. A temperature-dependent snowfall measure was adopted due to the lack of snow depth projections for the future periods.

The topographic variables were derived from the NASA Shuttle Radar Topography Mission (SRTM) digital elevation model (DEM) at a 30 arc-second spatial resolution^55,56^. The DEM was projected to a metric coordinate system (Equidistant cylindrical WGS84, ESRI: 54002) using bilinear resampling^57^ to calculate potential incident solar radiation (PISR) received by a land unit (ref.^58^, Equation 2 on p. 605) and slope gradient (Slope in Spatial Analyst tools). After the calculation, the resulting layers were re-projected to the geographical WGS 1984 system, again using bilinear resampling.

A modelled estimate for soil organic carbon content (SOCC, g kg^−1^) from SoilGrids1km data^59^ at a 30 arc-second resolution was used to account for the distinctive thermal properties (e.g., conductivity) of organic soils^60^. The data are not globally comprehensive because they do not cover non-vegetated areas. While it would be valid to assume a SOCC value of 0 for bare rock areas and deserts^59^, there are some zero-value areas, especially in the Canadian high Arctic, where a shallow organic cover may exist.

A global remote sensing based Water Bodies product (v 4.0), in a 150 m spatial resolution published by the ESA CCI’s (European Space Association Climate Change Initiative) Land Cover project^61^, allowed us to determine the amount of open water in the vicinity of MAGT or ALT site. The number of pixels representing water bodies were first summed (Aggregate in Spatial Analyst tools) inside each 30 arc-second grid cell, after which their proportional cover was calculated. Finally, modelling datasets were compiled assigning each MAGT and ALT observation with corresponding values of all environmental variables with grid cell-wise extraction (Extract Multi Values to Points in Spatial Analyst tools).

### Statistical modelling of the ground thermal regime

All statistical modelling was performed in the R environment (version 3.3.1.). Modelling techniques used were generalized additive models^62^ (GAM), generalized boosted models^63^ (GBM), generalized linear models^64^ (GLM) and random forest^65^ (RF). In the case of MAGT, we used an ensemble approach, in which medians of the predictions from these four statistical models were used to reduce uncertainties associated in the choice of a single method^66,67^. Due to the relatively low number of ALT observations, which with the ensemble approach could have caused overfitting and problematic extrapolation to future conditions, we chose to use only GLM predictions.

GAM was implemented in the R package *mgcv*^68^ with smoothing function k limited at value 3. The *gbm.step* function in the R package *dismo*^69^ with 10-fold internal cross-validation was used to fit GBM models with Gaussian error assumption for 10,000 trees at maximum. Further settings were set as follows: bagging fraction 0.75, learning rate 0.01 and interaction depth of six. GLM was calibrated using normal-error distribution and identity-link function. In the case of MAGT, predictors with first and second order polynomial terms were included, whereas only linear predictors were selected for ALT modelling. Random forest was implemented in the R package *randomForest*^70^. For each tree, four predictors were randomly selected to define optimal split in relation to MAGT and ALT. This was repeated to build a forest of 500 regression trees at maximum with a minimum node size of five.

The models were calibrated using TDD, FDD, precipitation sums above and below 0 °C, PISR and soil organic carbon content as predictors (Table 1). Water bodies were only considered in the MAGT models due to low amount of variation of water cover in the ALT dataset. Whenever predicted MAGT was ≤ 0 °C, a grid cell was considered to represent suitable conditions for permafrost occurrence. Hence, no distinction between continuous, discontinuous, sporadic or isolated permafrost was made. A detailed description of the employed modelling methods in a similar context is presented in our related work^22^.

### Settlement index (I_s_)

**Figure 3:**
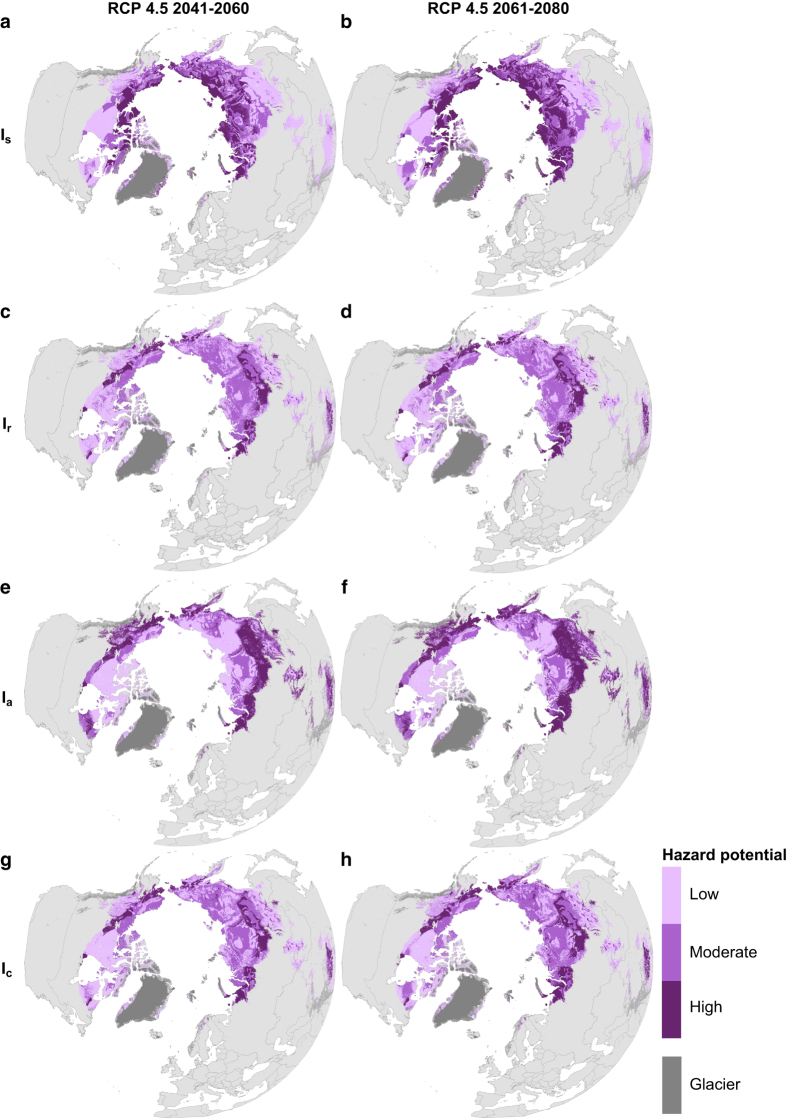
Geohazard indices in an orthographic projection showing near-surface permafrost degradation related risks to infrastructure. Displayed here, in a moderate Representative Concentration Pathway (RCP) 4.5 scenario for 2041-2060 and 2061–2080, are settlement index I_s_ (**a**,**b**, refs^71,72^), risk zonation index I_r_ (**c**,**d**, ref.^75^), AHP (analytic hierarchy process based index) I_a_ (**e**,**f**) and consensus of the three I_c_ (**g**,**h**). Each index consists of three mutually exclusive classes delimiting areas of low, moderate and high hazard potential. World Borders dataset is distributed under CC BY-SA 3.0 license (https://creativecommons.org/licenses/by-sa/3.0/) on http://thematicmapping.org/downloads/world_borders.php.

A settlement index^71,72^ (I_s_, Fig. 3a,b) was developed to present ‘a generalized geographic perspective on the potential effects of permafrost degradation on human infrastructure and activities’ (ref.^72^, p. 206). We repeated the formulation using volumetric ground ice content (GIC) data from the corresponding source^73^ and ALT-modelling results from this study. Prior to index computation, gridded (12.5 km resolution) GIC data were re-projected and classified into three classes: low = 0–10%, medium = 10–20% and high = over 20%, representing volumetric GIC. For computational purposes, class-mean values were specified for each class; 5, 15 and 35% respectively. In addition, the data make an overburden-thickness-based distinction between lowland and mountainous areas with ‘high to medium’ class (>10%), which we merged in the class of medium ice content (15%).

Wherever our modelled permafrost extent exceeded that of coarse-resolution GIC data (mainly along certain coastlines), we manually extrapolated the GIC value from the adjacent grid cell. In Iceland, our modelled permafrost extent was considerably more detailed. We assumed areas outside the GIC data to have relatively low ice content, considering their mountainous setting with thin soil and inherently low water content due to more rapid drainage. Similarly, a few isolated mountainous permafrost occurrences outside the GIC data were classified low in ice content if adjacent GIC values did not suggest otherwise.

Relative change (%) in ALT was calculated between the predictions of ALT in baseline (2000–2014) conditions and six future scenarios, namely RCP2.6, RCP4.5 and RCP8.5 for periods 2041–2060 and 2061–2080. Before computing the relative change, predicted ALT = 0 cm in a relatively few grid cells (5.8% of permafrost pixels in baseline) were forced to 1 cm to avoid dividing by zero and subsequent ‘no-data’ areas.

I_s_ computation employed two variables (Equation 1):

(1)Is=ΔZALT×Vice,

where ∆Z_ALT_ is the relative increase of ALT and V_ice_ the volumetric proportion of near-surface ground ice. Following ref.^72^, resulting values were logarithmically transformed and then reclassified into three classes by a nested means procedure^74^ with the two lower classes combined for a conservative estimate. The calculated class limits for the RCP2.6 in 2041–2060 period were implemented in classifying hazard values for each scenario to ascertain spatial and temporal between-scenario comparability.

### Risk zonation index (I_r_)

The second index is a permafrost degradation based risk zonation (I_r_, Fig. 3c,d)^75^. The zonation was originally computed exclusively for non-glacial land areas in Greenland at 25 km spatial resolution. In addition to ground ice data, their risk zonation involved soil properties and a ground-temperature-derived thermal criterion, and made a distinction between areas overlain by sediment and those of exposed bedrock. Assuming near-surface permafrost thaw has a negligible effect on bedrock in the context of engineering, they assigned bedrock ‘low risk’ areas. We used reclassified data on global soil thickness^76^ to determine areas without soil cover.

SoilGrids1km data^59^ were used to produce a two-class variable separating coarse and fine sediments with varying frost susceptibility – ‘low to negligible’ and ‘medium to very high’ respectively^75^. This was achieved by computing a mean value for volumetric silt and clay content (fine sediments) and sand content and coarse fragments (coarse sediments) at depth of 0–200 cm, and then determining which was dominant in a grid cell using a conditional expression in ArcMap Raster Calculator.

The concept of ‘Permafrost Thaw Potential’ (PTP) has been expressed as the potential ALT increase between the present time and a future scenario^75^. Whenever the increase exceeded 2.5 meters, PTP was classified high. In any other situation, low PTP resulted. For a surrogate for the PTP we used the MAGT predictions to simulate the thaw of near-surface permafrost. High PTP was assigned if a predicted negative baseline MAGT (indicating permafrost) rose above 0 °C for the scenario considered. In this way, both future periods under three RCPs were assigned with unique extents of thawing near-surface permafrost. Finally, we followed the decision-flow diagram^75^ to compile a four-class risk zonation. To comply with our other three-class indices, we merged the two classes of lowest risk (‘low risk’ and ‘limited risk’) together.

### Analytic hierarchy process based index (I_a_)

Analytic hierarchy process (AHP) is an efficient decision-making approach to examine complex problems, such as the specification of the relative roles of factors affecting natural hazards^77,78^. In AHP, it is necessary to: (1) determine the components of an unstructured problem (e.g., the factors affecting infrastructure hazards in the permafrost domain, such as ALT and slope gradient); (2) ordering these factors hierarchically (e.g., ALT is considered to be more important than slope angle); (3) give values (1–9) to subjective judgments based on the relative importance of each factor (see below); and (4) synthesize the decisions to define priorities to be assigned to these factors (e.g., computation of the weights of the selected factors). Consequently AHP requires a reciprocal pair-wise comparison matrix for the computation of weighted coefficients and geohazard index. The numerical values entered into the matrix are determined by comparing each factor based on a 9-point rating scale^79^. For example, if the factors are equally important for the final solution, a value of 1 is given, whereas 9 expresses the extreme importance of one factor over another^80^.

In our AHP analysis, we considered five environmental factors highlighted in the literature^14,81^: relative increase of ALT (see above); GIC; ground temperature (including near-surface permafrost thaw); fine-grained sediment content in the ground; and slope gradient. The relative importance of each variable was ranked using expert knowledge on permafrost conditions and engineering^80^. In this study, the ground temperature and thaw of near-surface permafrost was considered to be the most important factor affecting infrastructure damages followed by GIC, relative increase of ALT, fine-grained sediment content and slope gradient. Using the expert judgment (two experts) and a reciprocal pair-wise comparison matrix, weighted coefficients for each factor were computed (coefficients are shown in Equation 2). Due to the subjective nature of the pair-wise comparison, the quality of the results is dependent on the expert’s judgment. To assess the coherence of expert valuation a measure called the ‘consistency ratio’ was used to show the probability that the assessment matrix was randomly generated^79^. In a successful expert judgment^78^, the consistency ratio should be ≤ 0.1. Here, the consistency ratio was 0.09, indicating acceptable assessment.

To compute I_a_ (Fig. 3e,f), the five factors (ground temperature, GIC, ALT, fine-grained sediment content, and slope gradient) were first classified into three classes (note that the GIC was originally a three-class factor) based on their corresponding contribution to infrastructure hazard in permafrost environment^26^ (3 = high hazard, 2 = moderate hazard, 1 = low hazard). The ground temperature factor was formed by reclassifying the MAGT predictions. The highest hazard value was assigned to areas in which near-surface permafrost thaws (comparable to the PTP in risk zonation index). The areas where MAGT remains between −3 °C and 0 °C were considered moderate-hazard areas, whereas areas with MAGT below −3 °C represented the lowest hazard level^82^. Due to the lack of relevant threshold values, the nested-means approach^74^ was used to classify the numerically continuous ALT, fine-grained sediment, and slope variables to three-class factors. Fine-grained sediment content was derived from the SoilGrids1km data sets^59^ and slope gradient from the DEM^55,56^. The hazard potential of ALT, fine sediment and slope factors increased with increasing thaw depth, fine-grained sediment content, and slope inclination, respectively. We used the coefficients determined by comparison matrix and three-class raster layers to compute the AHP-based index (Equation 2):

(2)Ia=(groundtemperature×0.525)+(GIC×0.248)+(relativeincreaseofALT×0.122)+(fine-grainedsedimentcontent×0.071)+(slopegradient×0.035)

The resulting value of I_a_ ranged from 1.0 (lowest hazard potential) to 3.0 (highest hazard potential). To obtain a three-fold classification similar to the I_s_ and I_r_ we used the nested-means procedure^74^.

### Consensus of geohazard indices (I_c_)

Acknowledging the strengths and weaknesses associated with each index, a consensus method was employed to reduce uncertainty and to spotlight the most hazardous areas. We composed a consensus index (I_c_, Fig. 3g,h) by performing a majority vote procedure with ArcMap’s Cell Statistics tool. Wherever two or more indices shared a hazard potential value, this value was recorded to represent consensus. In draw situations, i.e., when all three had a different value a value of 2 was forced. The resulting high hazard classification was considered a strong indication of high risk for damage to infrastructure in a given location, whether the high hazard potential identified by separate indices was mainly due to deepening of the active layer (thaw settlement) or permafrost thaw at the ZAA depth (loss of bearing capacity/thaw settlement). All the indices were calculated for the area underlain by permafrost based on the predicted MAGT for the period 2000–2014. Owing to the patchiness of environmental variables used in hazard index formulations, minor differences in areal coverage existed between the indices. Gaps originated mainly from missing sediment-property data^59^ in certain high-Arctic islands and cold deserts. For the consensus index, we used the minimum overlapping extent of the three indices.

## Data Records

Data layers (n = 31, Table 2) for the maps of present and future permafrost extent and the geohazard indices (Data Citation 1) for each scenario are individually available in GeoTIFF format to enable reuse in various applications. The data are accessible through the PANGAEA, Data Publisher for Earth and Environmental Science (https://doi.org/10.1594/PANGAEA.893881).

## Technical Validation

### Statistical modelling evaluation

The predictive performances of the MAGT and ALT models were evaluated both with repeated random cross validation (CV) and against historical observational data (hindcasting). In CV, models were fitted 1,000 times with randomly sampled 95% of the data at each round and evaluated against the remaining 5%. To avoid the effects of spatial autocorrelation^83^, a distance constraint was set to omit calibration data observations closer than 500 km to observations in the evaluation data using function *zerodist2* in R-package *sp*^84^. The resulting dataset for each CV run had, on average, 450 MAGT and 150 ALT observations for calibration, and 50 MAGT and 25 ALT observations for evaluation. To evaluate model transferability, we compiled additional observational datasets (Supplementary Tables 1 & 2) applying the same criteria as with the baseline data. The data for 1985–1999 consisted of 250 MAGT and 155 ALT observations, and 1970–1984 had 253 and 16 observations, respectively.

The ensemble median of four modelling techniques for MAGT resulted in an adjusted coefficient of determination (R^2^) of 0.95 under baseline conditions (Table 3). The transferability of the models to past conditions was reasonable (R^2^ values 0.90 and 0.93) for the hindcasting periods 1970–1984 and 1985–1999, respectively. For ALT, the corresponding figures were 0.37 for current conditions and 0.57 for 1985–1999. The earlier period was not tested because there were too few observations.

Uncertainty assessments were performed with a re-sampling procedure, where 1,000 predictions were produced for 100,000 randomly chosen pixels across the entire study domain north of 30°N. For ALT, the analyses were limited to the modelled permafrost regions (MAGT ≤ 0 °C) at each RCP scenario during both of the future periods. In the procedure, 70% of observations were randomly sampled without replacement at each round. Over the 100,000 repetitions, we calculated 95% prediction intervals for each pixel, of which the 95^th^ percentile was considered the uncertainty estimate^85^. Uncertainty showed anticipated but reasonably small increases between baseline and future conditions (Table 3). When evaluated with the hindcast datasets, uncertainty values did not increase indicating that the models were stable.

In terms of predicted total area of present-day permafrost (Table 3), our model was in good agreement with that produced by ref.^13^, where an observed relationship between air temperature and permafrost occurrence was established to provide a probabilistic estimate of 15.5 (12.0–18.2) × 10^6^ km^2^. Similar extents for combined continuous and discontinuous permafrost were reported in a process-based model simulation^86^, where the mean of 15 models provided an extent of 16.2 × 10^6^ km^2^ (the range of models estimates was 7.6–21.1 × 10^6^ km^2^). It should be noted that there were differences in the baseline periods for model calibration between these studies and ours, and that ref.^86^ focused only on near-surface permafrost within 3 meters of the surface.

## Usage Notes

### Uncertainties in modelling permafrost thaw

The statistical approach to ground thermal regime modelling adopted in this study offers advantages over mechanistic methods, but also has intrinsic limitations, which have been addressed in refs^22,87^. While the frequently used process-based models can produce physically realistic simulations of ground thermal properties, their large-domain applicability at high spatial resolution is diminished by a lack of suitable data needed to realistically represent relevant processes^10,13^. Our modelling approach, in turn, used statistical-empirical relationships to relate field observations (i.e., responses, here MAGT and ALT) to local environmental conditions (i.e., explanatory variables or predictors), which can be readily derived from global geospatial data. Although especially the MAGT model had excellent predictive performance, it should be acknowledged that our approach is data-driven and subject to the data limitations^22,87^.

Statistical models often require calibration with data representing current conditions and with an assumption that the variables employed (apart from the climate projections) will remain in an equilibrium state in the future. Therefore, owing to the lack of applicable future projections, we excluded vegetation and land use from the analyses despite their multiple roles in permafrost dynamics^14,60^. This omission is likely to have added uncertainties in resolving local MAGT and ALT variability. Our approach is applicable in a predictive context because the focus was on near-surface permafrost that responds to climatic change relatively rapidly. It has been stated that it takes only several years for the temperature at DZAA to achieve a condition of quasi-equilibrium with prevailing climate conditions^81^. Hence, ground temperatures evolve with changing conditions.

Another source of uncertainty is related to limitations in gridded climate and soil data. The climate variables were based on interpolation of weather station data^51^. However, the weather stations (n = 24,542) have relatively good global coverage and the average errors between the observed and the interpolated values were small, < 0.3 °C for air temperature and mostly < 5 mm for precipitation^51^. Deriving snowfall predictor from climate data is, to a degree, an oversimplified way to assess snow thickness at local scale. Apart from uncertainties in interpolated climate surfaces (lower quality in sparsely sampled regions^51^), this is attributed to sub-pixel scale processes acting at a scale finer than our analysis, such as wind redistribution and the effects of microtopography and vegetation, which could not be accounted for. Moreover, although the global SoilGrids1km grids employed in this study clearly reproduce the spatial patterns of soil property variability, they are unable to explain most of its fine-scale spatial variation^59^. The accuracy of soil variables is likely to be lower in sparsely sampled regions, such as Siberia and northern Canada^59^. In addition, SOCC does not explicitly represent the thickness of organic horizons, which constitute an important component of the thermal offset.

To more realistically explain the fine-scale variability in the ground thermal regime, (especially in ALT, known to depend strongly on local hydrological and topographical conditions), an even finer resolution can prove useful^88^, as could consideration of soil texture and moisture and vegetation properties^89^. Despite the high modelling resolution, small patches of permafrost in peatlands may not have been captured by our modelling, owing to limitations in SOCC data.

Physical issues that cannot be fully addressed with geospatial data or parameterization also complicate the response. Predicting permafrost dynamics is complicated by its likely indirect spatial and temporal responses to changing climate, owing to the complexity of permafrost environments. Responses involve both negative and positive permafrost-ecosystem feedbacks that regulate permafrost by making it more resilient or vulnerable to warming climate^14,60,90^. One study^91^, for example, suggested that changing vegetation in a warming climate might result in a thicker organic layer, which could provide additional resilience to thaw through improved insulation of permafrost.

The initial state and thermal properties of permafrost also have important influences on its response to warming air temperatures^92^. Where permafrost temperature is close to 0 °C, the latent heat required to effect phase change from ice to water lowers the rate at which permafrost thaws^81^, especially in fine-grained material^93,94^. Cold permafrost, in turn, is more responsive to changes in air temperature owing to the lack of phase-change effects and a usually thinner buffering cover of vegetation and snow^15,95^. The thermal inertia of thick cold permafrost may, however, curtail warming although one circumpolar study^96^ did not find this effect to be substantial. The latent heat effect is amplified in conditions where ground ice content is high^97^. Any lag of permafrost response to changing climate is assumed to be most pronounced under the RCP8.5 scenarios, in which air temperature changes very quickly^13^.

### Geohazard indices

Our hazard potential maps show substantial geographic variation between the indices. This was expected, because each index considers at least some different hazard-affecting factors. The settlement index (I_s_), for example, highlighted the role of relative increases in ALT. Minor future increases in thaw, especially in the high-Arctic where only shallow annual thaw occurs, translated into large relative changes and therefore elevated thaw settlement potential. In these cold areas, I_s_ presumably exaggerated the hazard potential. Indices imposing no (I_r_) or less ALT change (I_a_) are less sensitive to this phenomenon and more relevant for also assessing the loss of bearing capacity. The most suitable index for reuses is thus encouraged to be determined on case-by-case basis.

An interconnection between high relative ALT change and hazard potential was visible in both the original zonation^71,72^ and I_s_ (Fig. 3a,b). Comparison of the two projections for the middle of 21^st^ century revealed similar patterns of projected hazard potential, although our indices suggested more extensive high-hazard areas in coastal lowlands of the Russian Arctic. High hazard potential of these areas was, however, similarly pronounced in another settlement index reproduction^98^. Similar to the original implementations^71,72^, mountainous areas, where ‘degradation-proof’ bedrock is at or near the surface, had predominantly low hazard potential. Although original coarse-resolution analyses did not identify potential localized areas of high hazard within those areas^71^, an increase in hazard potential in intermontane lowlands can now be distinguished. Owing to the fact that our approach did not take into account causal factors^99^ affecting slope hazards, and also to their often small areal extent and episodic occurrence, the present mapping is not suitable for detecting rockfalls and debris flows. Increasing MAGT and ALT are, however, considered to contribute to rising geomorphic instability in mountainous areas^8,99^.

The risk zonation index (I_r_) displayed high-risk values at the ‘thawing fringe’ around the polar permafrost (Fig. 2), whereas areas with thin soil cover, mountainous areas in particular, had low hazard potential. The analytical hierarchy partitioning based index (I_a_), in turn, identified hazard potential in topographically rough areas owing to the factor of slope gradient in its calculation. When compared to hazard zonation for Alaska^26^, I_a_ indicated similar relatively low hazard potential for mountainous areas, e.g., the Brooks Range, and elevated potential in low-lying discontinuous permafrost areas, e.g., in parts of the Seward Peninsula and along stretches of the Yukon River.

The averaging effect of the consensus index (I_c_) is visible in a more balanced hazard zonation (Fig. 3g,h). Because it yields a moderate risk value in cases where all three indices produce differing values, it can smooth out the extremities in any single index. This was most prominent when making comparisons with I_s_, which was prone to produce high risk values in areas where the active layer is initially thin. Such extremely cold high-Arctic areas displayed extensive high-risk potential in I_s_, but only moderate risk in I_c_ when I_r_ and I_a_ were taken into account.

Areas assigned high hazard values by two or more indices retained their high hazard potential in I_c_. The circumpolar distribution of such areas corresponds with the thawing fringe visible in I_r_ (Fig. 3c,d), but was more detailed in I_c_, especially in mountainous areas whose hazardous potential was most evident in I_a_. Substantial projected thawing does not, however, necessarily translate into high hazard potential. For example, extensive parts of thawing permafrost in the Canadian Shield had mostly low hazard values. Local factors, such as GIC and soil grain size, play decisive roles in determining the consequences of near-surface permafrost degradation.

A key limitation of the hazard assessments is that the available geospatial data on circumpolar ground ice content^73^ have coarse resolution and cannot resolve fine-scale variation. Moreover, the current data do not draw a distinction between pore ice contents below soil void ratio and excess ice, which largely determine the effective amount of thaw settlement^8,9,71^. In this study, extrapolation of ground ice values to modelled permafrost areas not covered by it together with the coarse initial data resolution and the required reclassification, is likely to have caused additional uncertainty. However, at present, no other ground ice data with hemispheric extent is available, despite a pronounced need in cryospheric studies^31^.

Despite the high analysis resolution, resolving fine-scale variability of hazard potential was hindered by the data limitations. Our data products can be considered to be a step forward toward global-scale geohazard indices and infrastructure risk assessments that are locally applicable. In addition to more accurate data on ground ice, climate data with high spatial and temporal resolution are needed. Refining the spatial resolution of remote sensing-based snow cover and water equivalent products could provide a crucial improvement in representing snow conditions. Similarly, enhanced datasets of soil properties (texture and organic matter content) and vegetation configurations are urgently needed for development of high-resolution permafrost modelling and more applicable geohazard indices. Such indices are paramount for assessing the impacts of changing climate on individual communities and for their adaption^6^.

The presented permafrost maps and geohazard indices have considerable potential for near-future infrastructure risk assessments across multiple scales. Related to the limitations in geospatial data, the indices are not suitable for specific construction project or local-scale planning, but are useful for delineating broad areas of hazard potential within which more localized investigations can be conducted. Identification of low and high-hazard areas can aid selection of and decision-making about suitable areas for new development, and for mitigating foreseeable damage (e.g., with technical and engineering solutions) to existing infrastructure^26,100–102^. An example of a successfully targeted risk management project in northern Canada^103^ illustrates the potential benefits of this approach. Extensive multidisciplinary local surveys in Salluit, Canada resulted in construction potential maps that assisted policy makers and managers seeking to apply sustainable urban management practices in areas affected by permafrost. Proactive adaptation has also been found to be beneficial at regional scales. These principles can yield substantial reductions in expenditures for mitigating the effects of permafrost degradation^27,104^.

## Additional information

**How to cite this article**: Karjalainen, O. *et al*. Circumpolar permafrost maps and geohazard indices for near-future infrastructure risk assessments *Sci. Data*. 6:190037 https://doi.org/10.1038/sdata.2019.37 (2019).

**Publisher’s note**: Springer Nature remains neutral with regard to jurisdictional claims in published maps and institutional affiliations.

## Supplementary Material



Supplementary Tables

## Figures and Tables

**Table 1:** Geospatial datasets and their derivatives, explained in the context of implementation in this study.

Data source	Variable description	Used in	Original resolution
WorldClim^51^	Freezing degree days (FDD), °C-days	MAGT and ALT modelling	30 arc seconds
	Thawing degree days (TDD), °C-days	MAGT and ALT modelling	30 arc seconds
	Precipitation in snow, mm	MAGT and ALT modelling	30 arc seconds
	Precipitation in water, mm	MAGT and ALT modelling	30 arc seconds
GMFD, Version 2^52^	Climate parameters for 2000–2014	Climate data reanalysis	0.5 degrees
USGS DEM^55^	Potential incident solar radiation^58^ MJ cm^−2^ y^−1^	MAGT and ALT modelling	30 arc seconds
	Slope gradient, °	Hazard indices (I_a_)	30 arc seconds
ESA CCI^61^	Water Bodies, %	MAGT modelling	5 arc seconds
SoilGrids1km^59^	Soil organic carbon content, g kg^−1^	MAGT and ALT modelling	30 arc seconds
	Coarse sediment content, %	Hazard indices (I_r_, I_a_)	30 arc seconds
	Fine sediment content, %	Hazard indices (I_r_, I_a_)	30 arc seconds
Pelletier *et al.*^76^	Average soil and sediment deposit thickness, m	Hazard indices (I_r_)	30 arc seconds
ALT model results	Relative increase in ALT, %Δ	Hazard indices (I_s_, I_a_)	30 arc seconds
MAGT model results	Near-surface permafrost thaw potential, thaw/no thaw	Hazard indices (I_r_)	30 arc seconds
	Near-surface permafrost stability (3 classes, see Hazard index formulation/Analytical hierarchy process based index)	Hazard indices (I_a_)	30 arc seconds
IPA^73^	Volumetric ground ice content, %	Hazard indices (I_s_, I_r_ and I_a_)	12.5 * 12.5 km
GMFD (Global Meteorological Forcing Dataset), USGS (United States Geological Survey), DEM (digital elevation model), ESA (European Space Association), CCI (Climate Change Initiative project), MAGT, (mean annual ground temperature), ALT (active layer thickness) and IPA (International Permafrost Association). Hazard indices are abbreviated as follows: I_a_ (analytic hierarchy process based index), I_r_ (risk zonation index) and I_s_ (settlement index).			

**Table 2:** Naming for the gridded data layers.

Time period/scenario	Permafrost extent	I_s_	I_r_	I_a_	I_c_
RCP2.6 2041–2060	PF_2650.tif	Is_2650.tif	Ir_2650.tif	Ia_2650.tif	Ic_2650.tif
RCP4.5 2041–2060	PF_4550.tif	Is_4550.tif	Ir_4550.tif	Ia_4550.tif	Ic_4550.tif
RCP8.5 2041–2060	PF_8550.tif	Is_8550.tif	Ir_8550.tif	Ia_8550.tif	Ic_8550.tif
RCP2.6 2061–2080	PF_2670.tif	Is_2670.tif	Ir_2670.tif	Ia_2670.tif	Ic_2670.tif
RCP4.5 2061–2080	PF_4570.tif	Is_4570.tif	Ir_4570.tif	Ia_4570.tif	Ic_4570.tif
RCP8.5 2061–2080	PF_8570.tif	Is_8570.tif	Ir_8570.tif	Ia_8570.tif	Ic_8570.tif
2000–2014	PF_baseline.tif	NA	NA	NA	NA
RCP = Representative Concentration Pathways, PF = permafrost extent map, I_s_ = settlement index, I_r_ = risk zonation index, I_a_ = analytical hierarchy partitioning based index and I_c_ = consensus index.					

**Table 3:** Adjusted coefficients of determination (R^2^) between the observed and predicted values for mean annual ground temperature (MAGT) and active layer thickness (ALT) models in baseline and past conditions (hindcast).

Period	MAGT R^2^	MAGT uncertainty ( ± , °C)	ALT R^2^	ALT uncertainty ( ± , cm)	Permafrost extent with uncertainty (10^6^ km^2^, ± )
Hindcast (1970–1984)	0.90	0.76	—	—	—
Hindcast (1985–1999)	0.93	0.77	0.57	37	—
Baseline (2000–2014)	0.95	0.77	0.37	37	15.1 (13.0–17.2)
RCP2.6 (2041–2060)		0.85		38	10.0 (8.2–12.2)
RCP4.5 (2041–2060)		0.86		38	9.1 (7.5–11.2)
RCP8.5 (2041–2060)		0.90		39	8.0 (6.2–9.8)
RCP2.6 (2061–2080)		0.85		38	9.9 (8.0–12.0)
RCP4.5 (2061–2080)		0.90		39	8.0 (6.3–9.9)
RCP8.5 (2061–2080)		0.98		42	5.4 (3.6–7.3)
Uncertainty values were determined also for both future periods under three emission trajectories (RCP, representative concentration pathway). MAGT evaluation was performed for model ensemble predictions, whereas ALT evaluation was based on GLM (generalized linear modelling) alone. Uncertainty ranges for permafrost extents were calculated using uncertainty values from MAGT predictions.					
