# Supplementary material for: Circumpolar permafrost maps and geohazard indices for near-future infrastructure risk assessments
Source: Sci Data. 2019 Mar 12;6:190037. doi: 10.1038/sdata.2019.37 (PMC6413688; doi:10.1038/sdata.2019.37)
Supplement: Supplementary Tables [file sdata201937-s2.pdf]

Supplementary Information for Karjalainen et al. "Circumpolar permafrost maps and geohazard indices for near-future infrastructure risk assessments"

Supplementary Table 1 ………………………………………………………………………… p. 1–2

Supplementary Table 2 …………………………………………………………………………. p. 3

Supplementary Table 1. Data sources used to compile mean annual ground temperature (MAGT) datasets for periods 1970–1984, 1985–1999 and 2000–2014. The number of observations from each source and for each period are provided.

Data source	Data title	Link to the data	MAGT observations		
			2000-2014	1985-1999	1970-1984
GTN-P Database	**Biskaborn, B. K. *et al.*** The new database of the Global Terrestrial Network for Permafrost (GTN-P). *Earth Syst. Sci. Data* **7,** 245–259 (2016).	http://gtnpdatabase.org/boreholes	464	38	32
NSIDC (National Snow & Ice Data Center, Boulder, Colorado, USA)	**Paetzhold, R. F.** Monthly Summaries of Soil Temperature and Soil Moisture at Sites in China (accessed 23rd December 2015) (2003).	http://nsidc.org/data/ggd625	2		
	**Minkin, M. & Melnikov, E.** Borehole and environmental protection descriptive and numerical data, Yamal Peninsula, Russia, Version 1 (accessed 28th September 2015) (1998).	http://nsidc.org/data/ggd402		56	68
	**Taylor, A. Burgess, M. M., Allen, V. & Wilkinson, A.** Canadian Geothermal Data Collection: Deep permafrost temperatures and thickness of permafrost, Version 1 (accessed 6th October 2015) (1998).	http://nsidc.org/data/ggd503		11	31
	**Clow, G**. Permafrost Temperature Data from a Deep Borehole Array on the Arctic Slope of Alaska (accessed 9th October 2015) (2015).	doi:10.5065/D6N014HK		16	21
	**Oberman, N. G. & Kakunov, N. B.** Ground Temperatures from Deep Boreholes in the Ob River Valley, Russia (VK-1615 and ZS-124/124a) (accessed 2nd October 2015) **(**2004).	http://nsidc.org/data/ggd646		2	2
Roshydromet	**Sherstiukov, A.** Dataset of daily soil temperature up to 320 cm depth based on meteorological stations of Russian Federation. *RIHMI-WDC* **176,** 224–232 (2012).	http://meteo.ru/data/164-soil-temperature	91	100	99
Geological Survey of Canada	**Smith, S. L. Riseborough, D. W., Ednie, M. & Chartrand, J.** A Map and Summary Database of Permafrost Temperatures in Nunavut, Canada. Geological Survey of Canada, Open File 7393 (2013).	doi:10.4095/292615	50		
	**Crow, H. L., Good, R. L., Hunter, J. A., Burns, R. A., Reman, A. & Russell, H. A. J.** Borehole geophysical logs in unconsolidated sediments across Canada. Geological Survey of Canada, Open File 7591 (2015).	doi:10.4095/295753	46	25	
	**Smith, S. L. & Ednie, M.** Ground thermal data collection along the Alaska Highway easement (KP 1559-1895) Yukon, summer 2014. Geological Survey of Canada, Open File 7762 (2015).	doi:10.4095/295974	7		
	**Ednie, M., Chartrand, J., Smith, S. L., Duchesne, C. & Riseborough, D. W.** Report on 2012 field activities and collection of ground thermal and active layer data in the Mackenzie Corridor completed under Northwest Territories Science Licence #15053. Geological Survey of Canada, Open File 7416 (2013).	doi: 10.4095/292864	1		
	**Wolfe, S. A., Smith, S. L., Chartrand, J., Kokelj, S., Palmer, M. & Stevens, C. W.** Geotechnical Database and Descriptions of Permafrost Monitoring Sites Established 2006-10 in the Northern Mackenzie Corridor, Northwest Territories. Geological Survey of Canada, Open File 6677 (2010).	doi: 10.4095/287167	1		
NGDS (National Geothermal Data System, U.S. Department of Energy)	**Blackett, R.** Utah Temperature-Depth Log Compilation. Utah Geological Survey (2013).	http://search.geothermaldata.org/dataset/utah-temperature-depth-log-compilation	20		
	**Maine Geological Survey** Maine Well Headers (2014).	http://search.geothermaldata.org/dataset/maine-well-headers	19		
	**Kelley, S.** New Mexico Temperature-Depth Logs and Graphic Profiles. New Mexico Bureau of Geology & Mineral Resources (2011).	http://search.geothermaldata.org/dataset/new-mexico-temperature-depth-logs-and-graphic-profiles	14		
	**Virginia Division of Geology and Mineral Resources** Georgia Well Logs (2012).	http://search.geothermaldata.org/dataset/georgia-well-logs	11		
	**Curran, J., Rancan, H. & French, M.** New Jersey Well Logs. New Jersey Geological and Water Survey (2013).	http://search.geothermaldata.org/dataset/new-jersey-well-logs	10		
	**Czajkowski, J.** Washington Well Logs. Washington Division of Geology and Earth Resources, Department of Natural Resources (2012).	http://search.geothermaldata.org/dataset/washington-well-logs	8		
	**Virginia Division of Geology and Mineral Resources** Virginia Well Logs (2012).	http://search.geothermaldata.org/dataset/virginia-well-logs	6		
	**University of North Dakota** Temperature at Depth Database (2014).	http://geothermal.smu.edu/static/DownloadFilesButtonPage.htm	4		
	**Virginia Division of Geology and Mineral Resources** Georgia Borehole Temperatures (2011).	http://search.geothermaldata.org/dataset/georgia-borehole-temperatures	1		
	**Gosnold, W.** Nebraska Temperature-Depth Data and Profiles. University of North Dakota (2013).	http://search.geothermaldata.org/dataset/nebraska-temperature-depth-data-and-profiles	1		

	**Niewendorp, C. A.** Oregon Well Logs. Oregon Department of Geology and Mineral Industries (2012).	http://search.geothermaldata.org/dataset/oregon-well-logs	1		
	**Harrison III, W. B.** Michigan Well Log Observation Data. Western Michigan University - Geosciences Department (2012).	http://search.geothermaldata.org/dataset/michigan-well-log-observation-data	1		
	**Nevada Bureau of Mines and Geology** Nevada Borehole Temperatures (2014).	http://search.geothermaldata.org/dataset/nevada-borehole-temperatures	1		
NOAA (National Oceanic and Atmospheric Administration, U.S. Department of Commerce)	**Huang, S., Pollack, H. N. & Shen, P.-Y.** Temperature trends over the past five centuries reconstructed from borehole temperatures. *Nature* **403,** 756–758 (2000).	doi:10.1038/35001556	13		
FMI (Finnish Meteorological Institute)	**Finnish meteorological institute** (2016).		9		
NSF Arctic Data Center (formerly ACADIS)	**NSF Arctic Data Center** Network of Permafrost Observatories in Western Alaska. Arctic Data Center (accessed 5th October 2015) (2014).	doi:10.18739/A2D934	3		
Nordicana D, Centre for Northern Studies	**Allard, M., Sarrazin, D. & L'Hérault, E.** Borehole and near-surface ground temperatures in northeastern Canada, Version 1.3 (1988-2014) (accessed 22nd January 2016) (2015).	doi:10.5885/45291SL-34F28A9491014AFD	3		
Permafrost Laboratory (University of Alaska, Fairbanks)	**The Geophysical Institute Permafrost Laboratory** Site Information and Historical Data Access (accessed 26th January 2016) (2010).	http://permafrost.gi.alaska.edu/sites_list?order=field_site_latitude_value&sort=asc&title_op=contains&title=	2		
Publications	**Ødegård, R. S., Isaksen, K., Eiken, T. & Sollid, J. L.** MAGST in Mountain Permafrost, Dovrefjell, Southern Norway, 2001–2006. Ninth International Conference on Permafrost, At University of Alaska Fairbanks, USA, Volume in *Proceedings Volume 2*, Kane D.L. & Hinkel, K.M. (eds.). Institute of Northern Engineering, University of Alaska Fairbanks, ISBN 978-0-9800179-3-9, 1311–1315 (2008).		2		
	**Streletskiy, D. A. *et al.*** Permafrost hydrology in changing climatic conditions: seasonal variability of stable isotope composition in rivers in discontinuous permafrost. *Environ. Res. Lett.* **10**, 095003 (2015).		2		
	**Peter, M.** Modeling of permafrost temperatures in the Lena River Delta, Siberia, based on remote sensing products. Master thesis, University of Leipzig (2015).	hdl:10013/epic.45589	2		
	**Günther, F., Overduin, P. P., Makarov, A. S. & Grigoriev, M. N.** Russian-German Cooperation SYSTEM LAPTEV SEA. The Expeditions Laptev Sea - Mamontov Klyk 2011 & Buor Khaya 2012. *Berichte zur Polar- und Meeresforschung* **664**, (2013).		1		
PERMOS (The Swiss Permafrost Monitoring Network)	**Swiss Permafrost Monitoring Network** PERMOS Database (accessed 25th August 2016) (2016).	doi:10.13093/permos-2016-01		2	
Geological Survey of Norway (NGU)	**Geological Survey of Norway** The NORPERM Permafrost Database (accessed 4th February 2016) (2016).	http://geo.ngu.no/kart/permafrost_svalbard	1		
**TOTAL**			**797**	**250**	**253**

Supplementary Table 2. Data sources used to compile active-layer thickness (ALT) datasets for periods 1970–1984, 1985–1999 and 2000–2014. The number of observations from each source and for each period are provided.

Data source	Data title	Link to the data	ALT observations		
			2000-2014	1985-1999	1970-1984
GTN-P database	**Biskaborn, B. K. *et al.*** The new database of the Global Terrestrial Network for Permafrost (GTN-P). *Earth Syst. Sci. Data* **7,** 245–259 (2015).	http://gtnpdatabase.org/activelayers<br>https://www2.gwu.edu/~calm	203	105	15
Publications	**Wu, Q., Zhang, T. & Liu, Y.** Thermal state of the active layer and permafrost along the Qinghai-Xizang (Tibet) Railway from 2006 to 2010. *Cryosphere* **6,** 607–612 (2012).		23		
Geological Survey of Canada	**Smith, S. L., Chartrand, J., Duchesne, C. & Ednie, M**. Report on 2014 field activities and collection of ground thermal and active layer data in the Mackenzie Corridor, Northwest Territories. Geological Survey of Canada, Open File 7935 (2015).	doi:10.4095/296958			
	**Chartrand, J., Ednie, M., Smith, S. L., Duchesne, C. & Riseborough, D. W.** Report on 2013 field activities and collection of ground thermal and active layer data in the Mackenzie Corridor. Geological Survey of Canada, Open File 7659 (2014).	doi:10.4095/295596			
	**Ednie, M., Chartrand, J., Smith, S. L., Duchesne, C. & Riseborough, D. W.** Report on 2012 field activities and collection of ground thermal and active layer data in the Mackenzie Corridor completed under Northwest Territories Science Licence #15053. Geological Survey of Canada, Open File 7416 (2013).	doi: 10.4095/292864			
	**Ednie, M., Chartrand, J., Smith, S. L., Duchesne, C. & Riseborough, D. W.** Report on 2011 Field Activities and Collection of Ground Thermal and Active Layer Data in the Mackenzie Corridor Completed Under Northwest Territories Science Licence #14918; Geological Survey of Canada, Open File 7231 (2012).	doi:10.4095/291982	21		
	**Ednie, M., Chartrand, J. & Smith, S. L.** Report on 2010 Field Activities and Collection of Ground Thermal and Active Layer Data in the Mackenzie Corridor Completed Under N.W.T. Science Licence #14686. Geological Survey of Canada, Open File 6932 (2011).	doi:10.4095/288924			
	**Smith, S. L., Throop, J., Ednie, M., Chartrand, J, Riseborough, D. & Nixon, F. M.** Report on 2009 field activities and ground thermal data collection in the Mackenzie Corridor completed under N.W.T. science licence #14582. Geological Survey of Canada, Open File 6695 (2010).	doi:10.4095/287166			
	**Smith, S.L., Riseborough, D. W., Nixon, F. M., Chartrand, J., Duchesne, C. & Ednie, M.** Data for Geological Survey of Canada active layer monitoring sites in the Mackenzie Valley, N.W.T.; Geological Survey of Canada, Open File 6287 (2009).	doi:10.4095/248197	17	48	
	**Wolfe, S. A. *et al.*** Report on 2010-11 permafrost investigations in the Yellowknife area, Northwest Territories. Geological Survey of Canada, Open File 6983 (2011).	doi:10.4095/289596	5		
	**Leblanc, A.-M. *et al.*** Assessing permafrost conditions and landscape hazards in support of climate change adaptation in Pangnirtung, Nunavut. Geological Survey of Canada, Open File 6868 (2011).	doi:10.4095/289548	4		
LTER (Long Term Ecological Research, University of Alaska, Fairbanks)	**Ruess, R. W. & Hollingsworth, T. N.** Active Layer Depth or Permafrost Presence for the Regional Site Network. Bonanza Creek LTER - University of Alaska Fairbanks. BNZ:605 (accessed 14th March 2016) (2015).	doi:10.6073/pasta/384e147c68e6d13dffc8d6cec2cb6312	20		
PERMOS (The Swiss Permafrost Monitoring Network)	**Swiss Permafrost Monitoring Network** PERMOS Database (accessed 12th March 2016) (2016).	doi:10.13093/permos-2016-01	9		
NSIDC (National Snow & Ice Data Center, Boulder, Colorado, USA)	**Rönkkö, M.** Active-Layer Depth of a Finnish Palsa Bog (accessed 15th March 2016) (2003).	http://nsidc.org/data/ggd622	1	1	
	**Thórhallsdóttir, T.E.** Seasonal and annual dynamics of frozen ground, central highland of Iceland, Version 1 (accessed 6th October 2015) (1998).	http://nsidc.org/data/ggd9		1	1
**TOTAL**			**303**	**155**	**16**
